# Injectable Hydrogel Based on Modified Gelatin and Sodium Alginate for Soft-Tissue Adhesive

**DOI:** 10.3389/fchem.2021.744099

**Published:** 2021-09-22

**Authors:** Yuhang Xing, Xueqin Qing, Hao Xia, Shiqi Hao, Haofang Zhu, Yiyan He, Hongli Mao, Zhongwei Gu

**Affiliations:** ^1^ Research Institute for Biomaterials, Tech Institute for Advanced Materials, College of Materials Science and Engineering, Nanjing Tech University, Nanjing, China; ^2^ Department of Pediatrics, Shanghai General Hospital, School of Medicine, Shanghai Jiao Tong University, Shanghai, China; ^3^ NJTech-BARTY Joint Research Center for Innovative Medical Technology, Nanjing, China; ^4^ Suqian Advanced Materials Industry Technology Innovation Center of Nanjing Tech University, Nanjing, China

**Keywords:** soft-tissue adhesive, sodium alginate, gelatin, Schiff base reaction, hydrogel

## Abstract

To assist or replace the traditional suture techniques for wound closure, soft-tissue adhesives with excellent adhesion strength and favorable biocompatibility are of great significance in biomedical applications. In this study, an injectable hydrogel tissue adhesive containing adipic acid dihydrazide–modified gelatin (Gel-ADH) and oxidized sodium alginate (OSA) was developed. It was found that this tissue adhesive possessed a uniform structure, appropriate swelling ratio, good injectability, and excellent hemocompatibility and cytocompatibility. The adhesion capacity of the developed adhesive with optimized component and concentration was stronger than that of the commercial adhesive Porcine Fibrin Sealant Kit. All these results suggested that the developed hydrogel was a promising candidate for a soft-tissue adhesive.

## Introduction

Rapid hemostasis and effective wound closure are very important in wound repair. Conventional suturing is usually time consuming and cannot close the wound immediately, which can lead to a variety of pathologic scenarios, such as tissue morbidities and mortalities (Geng et al., 2020; Zhu et al., 2020). Sutureless wound closure, which primarily utilizes medical adhesives, shows adequate ability in wound sealing and healing with less pain and scars (Gowda et al., 2020; Zhou et al., 2021). In addition, compared to conventional suture, it is more acceptable by the patient. Therefore, there are different types of medical adhesives have been developed in recent years. However, most of the available adhesives have certain limitations. For example, fibrin glue has favorable biocompatibility and can facilitate wound repair, but it cannot be used alone because of its unreliable mechanical and adhesion capacity. Besides, it is rather expensive and may lead to a risk of blood-borne disease or virus infections (Du et al., 2020; Gao et al., 2020). In comparison with fibrin glue, cyanoacrylates have much stronger adhesion capacity, but the excessive heat and the cytotoxic byproducts produced in the fast polymerization process during bonding are a major concern (Lv et al., 2021). Additionally, their poor elasticity cannot accommodate the movement of soft tissues under a highly dynamic physiological environment (Li et al., 2020; Zhao et al., 2021). Therefore, development of ideal medical adhesives with good biocompatibility, sufficient mechanical property, tunable biodegradability, and rapid and strong adhesion has a great application value in wound closure and healing (Neuffer et al., 2004; Gillman et al., 2020).

Herein, by mimicking the composition of the extracellular matrix (ECM) of native tissues, a strategy to prepare adhesives with good biocompatibility and multiple functions, from polypeptide and polysaccharide, was proposed (Yang et al., 2017; Ke et al., 2020). Firstly, gelatin was modified with adipic acid dihydrazide (Gel-ADH) and sodium alginate was oxidized (OSA) with NaIO_4_. Then, the hydrogel adhesive was prepared through the Schiff base reaction between Gel-ADH and OSA. The results showed that the Gel-ADH/OSA hydrogel had an appropriate swelling ratio, good injectability, excellent biocompatibility, and improved adhesion capacity.

## Materials and Methods

### Materials

Sodium alginate (low viscosity), gelatin (from porcine skin), and sodium periodate (NaIO_4_) were purchased from Aladdin Industrial Corporation (Shanghai, China). Dulbecco’s modified Eagle medium (DMEM) and fetal bovine serum (FBS) were purchased from Life Technologies Corporation (California, United States). A Porcine Fibrin Sealant Kit was purchased from Guangzhou Bioseal Biotech Co., Ltd. All other chemicals were purchased from Sigma-Aldrich and used as received unless specified otherwise.

### Preparation of OSA

Oxidized sodium alginate was synthesized as reported previously (Yuan et al., 2018). Briefly, 3.00 g raw sodium alginate (SA) was dissolved in ultrapure water (300 ml) and 4.86 g NaIO_4_ was added. After that, the mixed solution was stirred for another 4 h without light, and then 5 ml ethylene glycol was added in the mixed solution to terminate the oxidation. The final product was dialyzed (MWCO = 3,500 Da) for 3 days in distilled water. Finally, the product was lyophilized. To confirm the characteristic peak of the aldehyde group in OSA, a Fourier transform infrared (FTIR) spectroscope (Nicolet IS 10, Thermo Scientific, United States) was used and the measurement was performed at room temperature and recorded in the 4,000–400 cm^−1^ range.

### Preparation of Gel-ADH

To synthesize ADH-modified gelatin, 3.00 g gelatin and 2.40 g adipic acid dihydrazide were dissolved in 300 ml ultrapure deionized water with stirring at 55°C. Then, 0.50 g EDC and 0.50 g HOBt were added to the solution. Finally, 0.1 M HCl solution was used to adjust the pH of the abovementioned solution to 5. The mixed solution was stirred overnight. The final product was dialyzed (MWCO = 7,000 Da) for 5 days in distilled water (Hozumi et al., 2018). The ^1^H NMR spectra of the Gel-ADH was measured by using a 600 Hz NMR spectrometer (JNM-ECZR-600 Hz, JEOL, United States )

### Degree of Oxidation Study

The degree of oxidation was obtained by the titration method (Reakasame and Boccaccini, 2018). In brief, W g OSA, 5 ml anhydrous ethanol, and 5 ml hydroxylamine hydrochloride solution were added in a flask. Then, the solution was heated to reflux for 3 h and left to set for at least another 2 h. When the solution was cooled to room temperature, 0.1 M NaOH was used to titrate the mixture. When the color of the mixed solution became yellow, the titration was stopped and the volume (V_1_) of the titrimetric NaOH solution was recorded immediately. Then, 5 ml anhydrous ethanol and 5 ml hydroxylamine hydrochloride solution were added to another flask. The previous steps were repeated and the volume (V_2_) of titrimetric NaOH solution was recorded. The oxidation degree (OD) was calculated by the following Equation:

$$\begin{array}{c} OD\left(\%\right)=198\times 0.1\times \frac{V_{1}-V_{2}}{2W}\times 100 \end{array}.$$ (1)

### Preparation of Gel-ADH/OSA Hydrogels

Lyophilized OSA and Gel-ADH were dissolved in the PBS solution (pH = 7.4). The prepolymer concentration was 10, 15, and 20% (w/v), respectively. The Gel-ADH and OSA solution were added to a glass bottle to prepare the four types of hydrogels. The volume ratio of the Gel-ADH and OSA solution was 1:1 and 2:1, respectively. The prepared hydrogels were represented as 10% Gel-ADH/OSA 1:1, 15% Gel-ADH/OSA 1:1, 20% Gel-ADH/OSA 1:1, and 20% Gel-ADH/OSA 2:1 accordingly.

### Gelation Time of the Gel-ADH/OSA Hydrogel

Gelation time was measured at room temperature by tilt tests. Briefly, four abovementioned types of mixed solutions were injected into glass test tubes immediately after preparation. The tube was tilted horizontally every second, and then, the time when the solution did not have any flow was recorded as the gelation time.

### Swelling Behavior of the Gel-ADH/OSA Hydrogel

The hydrogel samples were lyophilized and immersed in PBS (pH = 7.4). The initial weight of the lyophilized hydrogel was recorded as *W*_*0*_. After 12 and 24 h, the hydrogel samples were taken out from the PBS solution and the PBS on the surface of the hydrogels was wiped with filter papers. The weight of the hydrogels at predetermined intervals was recorded as *W*_*t*_. The swelling ratio was calculated as follows:

$$\begin{array}{c} Swelling\;ratio\;\left(\text{\%}\right)=\frac{W_{t}-W_{0}}{W_{0}}\times 100\;\% \end{array}.$$ (2)

### Micromorphology of the Gel-ADH/OSA Hydrogel

The microstructure of the lyophilized Gel-ADH/OSA hydrogel was observed by SEM (XL30 ESEM, Philips, Netherlands). The freeze-dried hydrogels were put on a platform and coated with a gold layer. Then, the cross-sectional morphology images were observed by SEM.

### Hemolytic Activity of the Gel-ADH/OSA Hydrogel

The erythrocytes were extracted from the fresh mouse blood by centrifugation (1,000 rpm, 5 min) and diluted to 5% (v/v) with PBS solution (pH = 7.4). The prepared four types of hydrogels were mixed with the erythrocytes solution and incubated for 3 h (37°C). Finally, the mixtures were centrifuged at 2,500 rpm for 10 min and the absorbance of the supernatant from each tube was obtained by using a microplate reader (MK3, Thermo, United States ) at 540 nm. The percentage of hemolysis was calculated as follows:

$$\begin{array}{c} Hemolysis\;\left(\text{\%}\right)=\frac{OD_{\text{m}}-OD_{p}}{OD_{\text{w}}-OD_{p}}\times 100\;\% \end{array},$$ (3)

where *OD*_*p*_ *, OD*_*w*_ *,* and *OD*_*m*_ were the absorbance of the supernatant obtained from the erythrocytes mixture with PBS, ultrapure H_2_O, and prepared hydrogels, respectively. Cells treated with the PBS solution were used as a positive control, while cells treated with H_2_O were used as a negative control. The hemolytic activity was measured in triplicate.

### Cytocompatibility of the Gel-ADH/OSA Hydrogel

The cytocompatibility of the hydrogels to L929 cells was evaluated by the CCK-8 method (Zhang et al., 2020). Briefly, the cells were incubated in DMEM supplemented with 10% FBS and 1% penicillin–streptomycin solution at 37°C with 5% CO_2_. The four abovementioned types of hydrogels were prepared in 96-well plates at 37°C, and the cells (5 × 10^3^ cells/well) were cultured with the hydrogels. After 1 and 3 days, 10 μl of CCK-8 was added to each well and incubated for another 2 h. Finally, the absorbance of each well was obtained by using a microplate reader (MK3, Thermo, United States ) at 450 nm. The hydrogel-free DMEM was used as a control. The viability of cells was measured in triplicate.

### Rheological Analysis

The rheological measurements of the hydrogels were conducted by using a rheometer (MCR 302, Anton Paar, Austria) at 25°C. A 20% Gel-ADH/OSA 1:1 hydrogel was placed on a 25 mm diameter parallel plate of the rheometer. A shear-thinning test using the Gel-ADH/OSA adhesive was measured with a shear rate in the range of 0–200 1/s.

### Adhesive Capacity of the Gel-ADH/OSA Hydrogel

A tissue adhesion capacity test was performed using fresh porcine skins. The porcine skins were immersed in PBS (pH = 7.4) overnight before use. Then, the porcine skins were cut into two oblong samples (6 cm × 1.5 cm). A 200 μl of Gel-ADH solution was applied on the first porcine skin, and 200 μl of OSA solution was applied on the second porcine skin. After that, two porcine skins were overlapped and the contacting area was 2 cm × 1.5 cm. Finally, the porcine skins were loaded 50 g for 5 min. The adhesion capacity (kPa) was recorded with a universal tester at a rate of 10 mm/min.

### Statistical Analysis

All samples were measured in triplicate, and the data were represented as mean ± standard deviation (SD). All data were analyzed by one-way analysis of variance (*ANOVA*), and a value of *p* < 0.05 was considered to be statistically significant.

## Results and Discussion

### Synthesis and Characterization of OSA and Gel-ADH

**FIGURE 1:**
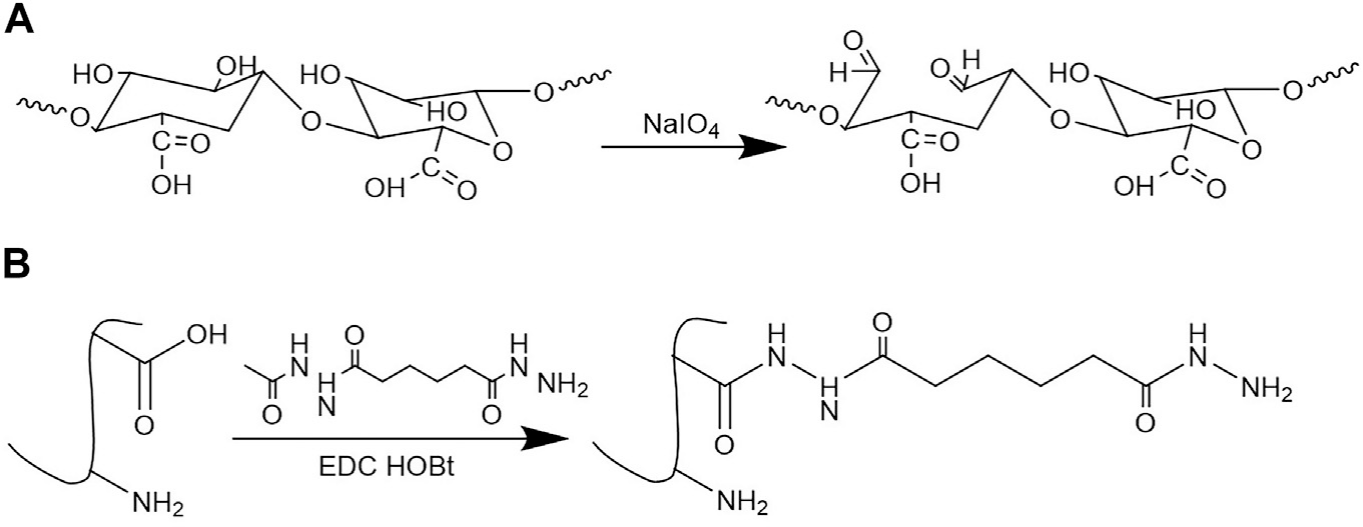
Synthesis of **(A)** oxidized sodium alginate (OSA) and **(B)** adipic acid dihydrazide–modified gelatin (Gel-ADH).

**FIGURE 2:**
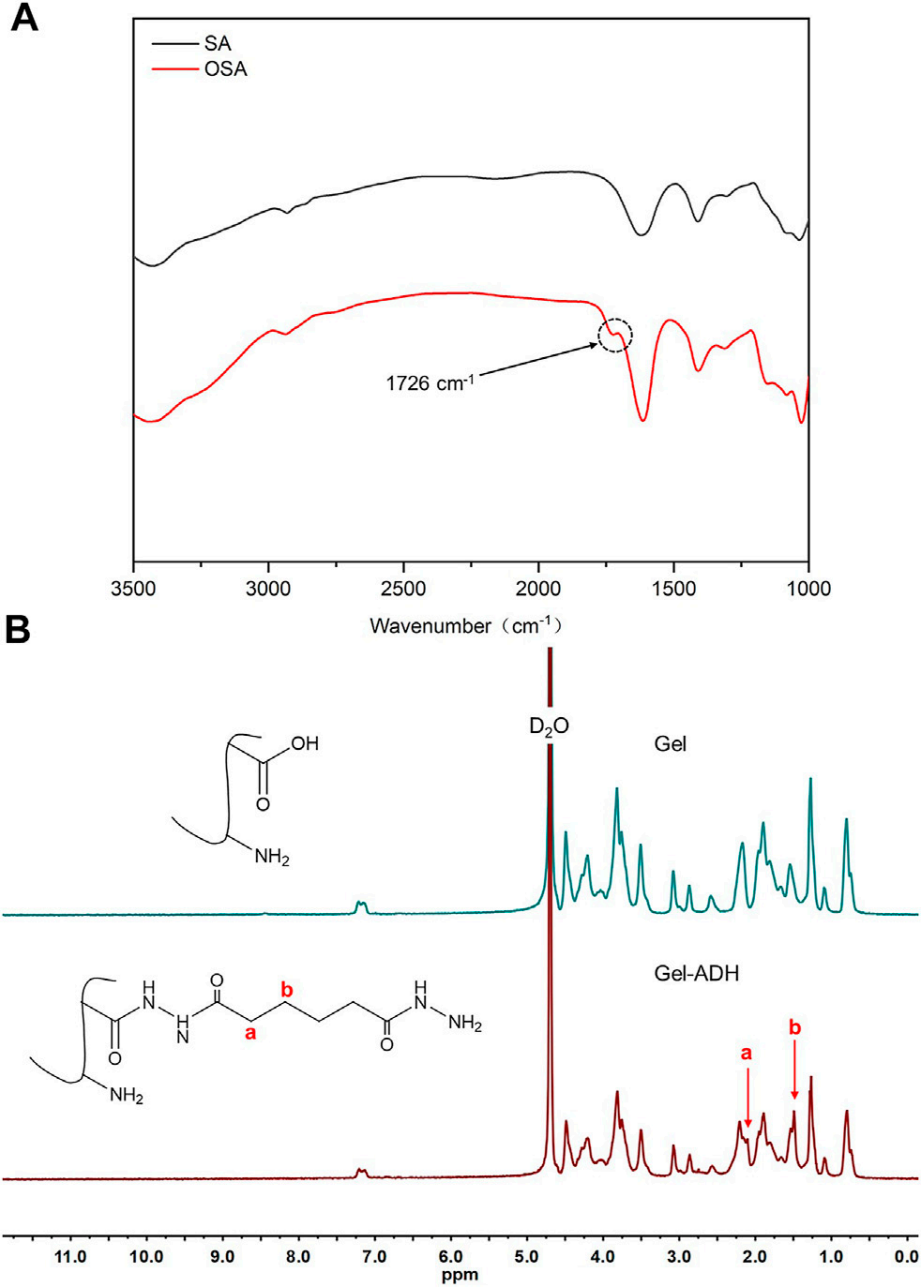
**(A)** FTIR spectra of SA and OSA. **(B)** ^1^H NMR spectra of Gel and Gel-ADH.

The preparation scheme of OSA and Gel-ADH is shown in Figure 1. Figure 2A shows the FTIR spectra of SA and OSA. Compared with the spectra of SA, a new absorption peak at 1726 cm^−1^ which is due to the aldehyde group was observed in OSA. The oxidation degree of OSA used in this study was about 79%. The ^1^H NMR spectra of Gel and Gel-ADH are shown in Figure 2B. Compared with the curve of Gel, new methylene peaks of ADH at 2.07 and 1.46 ppm were found in the Gel-ADH, indicating that gelatin was successfully modified with adipic acid dihydrazide. The degree of substitution (DS) of ADH, defined as the number of ADH moieties per 100 carboxyl groups of gelatin, was determined from the ^1^H NMR spectra (Figure 2B) by comparing the integrals of signals at 1.46 (A_1.46_) and 2.60 ppm (A_2.60_). DS, which was expressed as A_1.46_/(A_1.46_ + A_2.60_) × 100% (Yan et al., 2017), was calculated to be 67%.

### Characterization of the Gel-ADH/OSA Hydrogel

**FIGURE 3:**
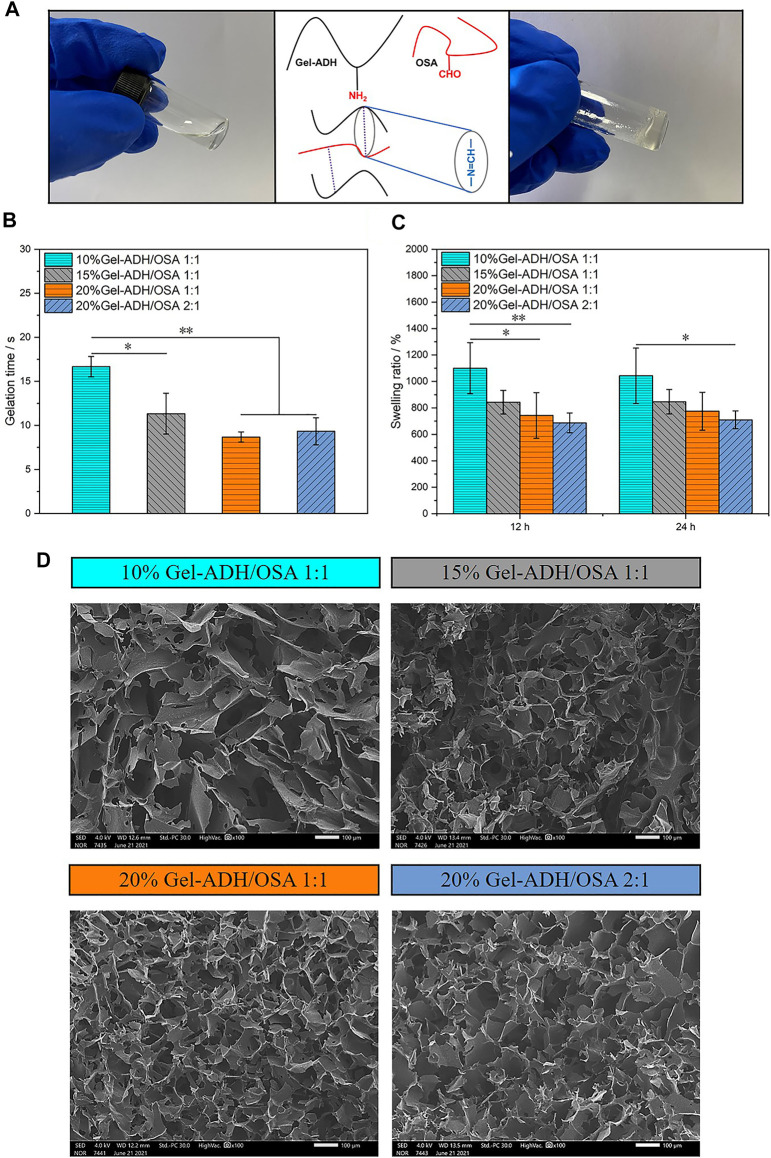
**(A)** Schematic illustration of the preparation of Gel-ADH/OSA hydrogels. **(B)** Gelation time, **(C)** swelling ratio, and **(D)** SEM images of Gel-ADH/OSA hydrogels with different components and concentrations (scale bar = 100 μm, *p＜0.05, **p ＜ 0.01, *n* = 3).

Without further treatment, the sol-gel transition of the Gel-ADH/OSA hydrogel occurred relatively fast, as indicated by tilt tests (Figure 3A). Meanwhile, the hydrogel adhesive developed here was completely transparent and would not cover the underlying tissues when used by surgeons. Figure 3B shows the gelation time of the Gel-ADH/OSA hydrogel with different components and concentrations. The gelation time of 10% Gel-ADH**/**OSA 1:1, 10% Gel-ADH**/**OSA 1:1, 15% Gel-ADH**/**OSA 1:1, and 20% Gel-ADH**/**OSA 2:1 was 16.7, 11.3, 8.7, and 9.3 s, respectively. For wound sealing, the gelation time of the hydrogel adhesive can affect the hemostasis of damaged tissues. Some commercial tissue adhesives have strong adhesion capacity, but the gelation time is too slow, which may not be enough to stop bleeding in the event of a sudden loss of blood (Qiao et al., 2021). Here, the gelation time of 20% Gel-ADH/OSA 1:1 and 20% Gel-ADH/OSA 2:1 was under 10 s. The Gel-ADH/OSA hydrogel with higher concentration had more amino and aldehyde groups to accelerate the Schiff base reaction rate.

An appropriate swelling ratio is an important prerequisite for medical tissue adhesive to maintain a lasting and stable adhesion effect *in vivo* (Liang et al., 2021). If the swelling ratio is too high, the adhesive will squeeze the trauma area, which is not conducive to the adhesion of the wound. The swelling ratio of Gel-ADH/OSA adhesive is dependent on the prepolymer component and concentration. As shown in Figure 3C, with the increase of prepolymer concentration or the content of Gel-ADH, the swelling ratio decreased which may be caused by the increased internal cross-linking sites in the system. This result indicated that the dense network restricted the expansion of the hydrogel. The swelling ratio of 15% Gel-ADH/OSA 1:1, 20% Gel-ADH/OSA 1:1, and 20% Gel-ADH/OSA 2:1 was under 1,000%. However, the swelling ratio of 10% Gel-ADH/OSA 1:1 decreased after 24 h, probably because of its low concentration, resulting in faster rate of degradation. All these studies proved that Gel-ADH/OSA had good swelling ratio and excellent biodegradability.

Figure 3D shows the internal microscopic morphology of the lyophilized hydrogel characterized by scanning electron microscopy. With the increase of the Gel-ADH component and prepolymer concentration, the porosity decreased gradually. Meanwhile, a uniform microstructure usually provides better mechanical properties theoretically for hydrogels (Pourjavadi et al., 2020). The network structure of Gel-ADH/OSA adhesive presented a high degree of homogeneity.

### Biocompatibility of the Gel-ADH/OSA Hydrogel *In Vitro*

**FIGURE 4:**
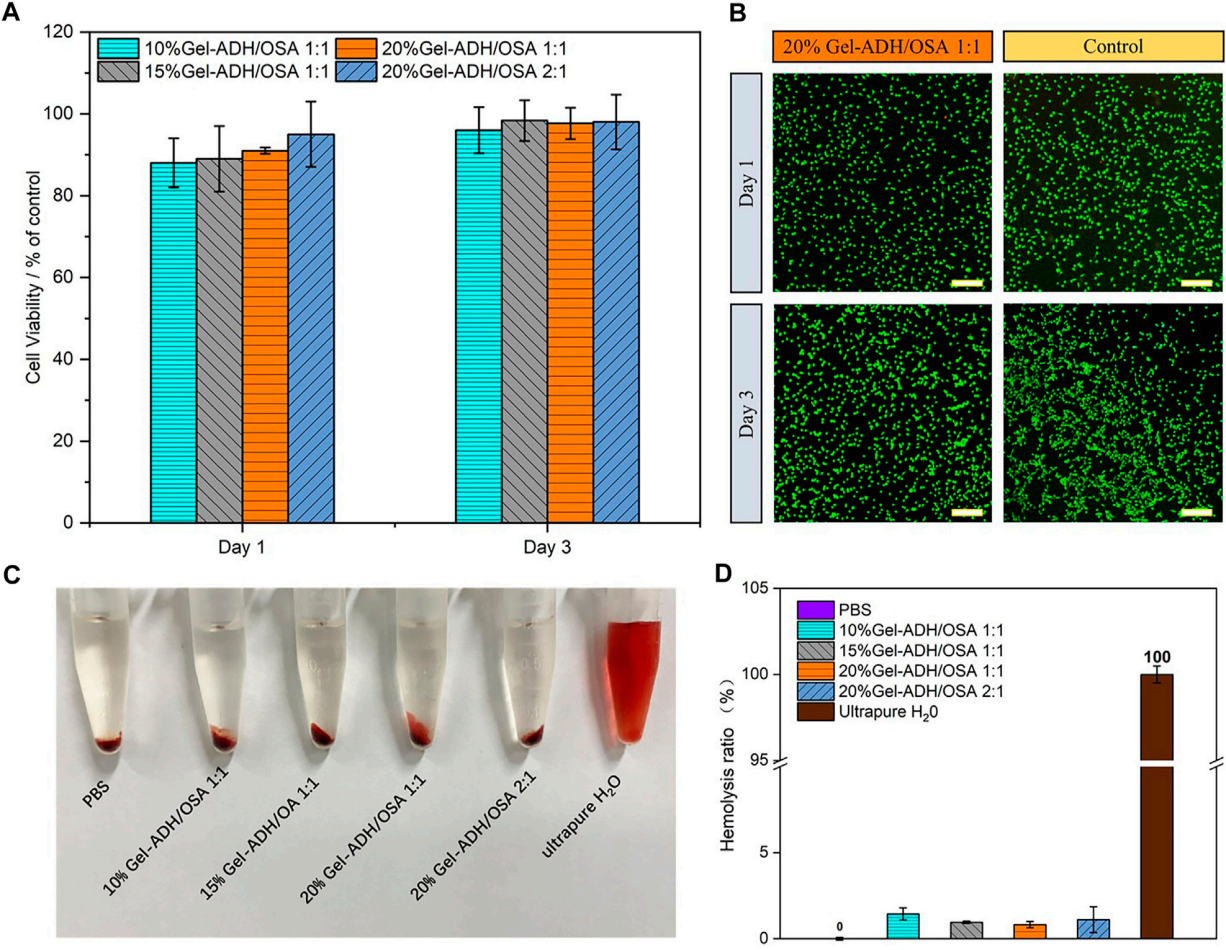
**(A)** Cell viability assay of L929 cells after being incubated with Gel-ADH**/**OSA hydrogels with different components and concentrations for 1 and 3 days **(B)** Live**/**dead staining of L929 cells after being incubated with the 20% Gel-ADH**/**OSA 1:1 hydrogel for 1 and 3 days (scale bar = 100 μm). **(C)** Image and **(D)** quantitative analysis of the hemolytic activity of Gel-ADH/OSA hydrogels with different components and concentrations.

It is well known that one of the key factors hindering the wide application of biomedical adhesives is their toxic and side effects on cells or their degradation products that can lead to wound tissue infection and necrosis. To investigate the biocompatibility of the Gel-ADH/OSA hydrogel, L929 cells were employed and directly incubated with the Gel-ADH/OSA hydrogel. Then, the CCK-8 assay and live/dead staining were performed on the first and third day of incubation (Figures 4A,B). Compared to that of the control group, cells cultured with Gel-ADH/OSA hydrogels with different compositions and concentrations showed a survival rate higher than 90% at both day 1 and day 3, indicating the hydrogels have no obvious negative effect on cell viability. This result was also consistent with the live/dead staining (Figure 4B). Most of the cells were stained green in all the groups.

Meanwhile, a hemolysis test was carried out to investigate the hemocompatibility of Gel-ADH/OSA hydrogels. The gross appearance of the erythrocytes after been treated with the solution of PBS, Gel-ADH/OSA hydrogels with different compositions and concentrations, and pure water is shown in Figure 4C. The solutions from Gel-ADH/OSA hydrogels were found to be light red, which was similar to those of the PBS group, while the water group showed bright red color. Figure 4D shows the quantitative analysis result of the hemolysis test, and the hemolysis ratios of Gel-ADH/OSA hydrogels were 0.82–1.44%, indicating Gel-ADH/OSA hydrogels have excellent hemocompatibility.

### Rheological and Tissue Adhesion Capacity of Gel-ADH/OSA Hydrogels

**FIGURE 5:**
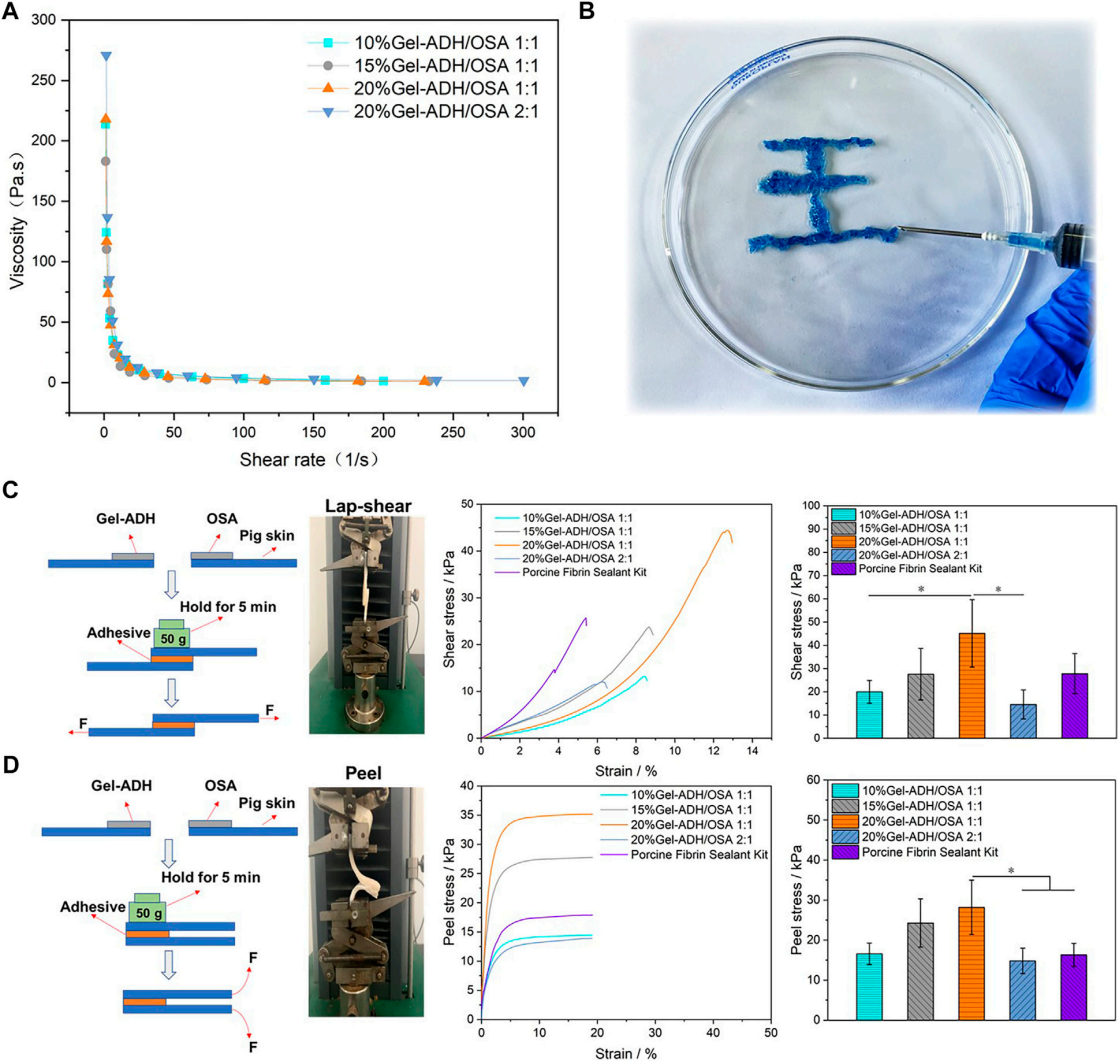
**(A)** Shear-thinning ability of Gel-ADH/OSA hydrogels with different components and concentrations. **(B)** Injectability of the 20% Gel-ADH/OSA 1:1 hydrogel. A schematic and quantitative analysis of **(C)** a standard lap shear test and **(D)** a standard peel test using Gel-ADH/OSA hydrogels with different components and concentrations. A commercialized Porcine Fibrin Sealant Kit served as the control (*p＜0.05, *n* = 3).

To check whether the Gel-ADH/OSA adhesive has a shear-thinning property, a static shear rate sweep was performed. As shown in Figure 5A, with the increase of shear rate, the viscosity of the hydrogel decreased rapidly. When the shearing rate was 15 1/s, the 10% Gel-ADH/OSA 1:1 and 20% Gel-ADH/OSA 2:1 had a low viscosity of 18 Pas. When the shearing rate was 18 1/s, the 15% Gel-ADH/OSA 1:1 and 20% Gel-ADH/OSA 1:1 had a low viscosity of 9 Pas. Therefore, the Gel-ADH/OSA adhesive was shear-thinning because of dynamic Schiff base covalent bonding. As shown in Figure 5B, the Gel-ADH/OSA adhesive had excellent injectability in the process of application (Yang et al., 2020).

To explore the adhesion capacity of Gel-ADH/OSA hydrogels *in vitro*, standard shear and peer tests were performed (Suneetha et al., 2019) (Figures 5C,D). As shown in Figure 5, with the increase of prepolymer concentration, the shear and peer stress of Gel-ADH/OSA hydrogels increased significantly. That is because the gelatin and sodium alginate are sticky inherently, and the Gel-ADH/OSA hydrogel with higher concentration had more amino and aldehyde groups to accelerate the Schiff base reaction rate. Meanwhile, the high oxidation degree of OSA would accelerate cross-linked reactions and improve the adhesive strength of the hydrogel. Since the 20% Gel-ADH/OSA 1:1 has more sites to react with amino groups on the surface of skin tissue, the lap shear strength (45 ± 14 kPa) and peel stress (28 ± 6 kPa) were higher than those of other groups, including the commercial adhesive Porcine Fibrin Sealant Kit. Previous studies also showed that the Fibrin Sealant has limited adhesion strength (Chow et al., 2021). To further confirm the biomedical application potential of the hydrogel adhesive, wound sealing and healing investigation will be performed in the future animal studies.

## Conclusion

In this study, an injectable hydrogel tissue adhesive was developed, and the hydrogel showed a suitable swelling ratio, good injectability, and excellent biocompatibility. We also found that the adhesion capacity was related to the prepolymer component and concentration. The 20% Gel-ADH/OSA 1:1 hydrogel showed the highest adhesion ability, which was also higher than that of the commercial Porcine Fibrin Sealant Kit. All these results suggested that the polysaccharide-based hydrogel was a promising candidate for a soft tissue adhesive.

## Data Availability

The original contributions presented in the study are included in the article/Supplementary Material; further inquiries can be directed to the corresponding authors.
